# Effect of Capsaicinoids on Neurophysiological, Biochemical, and Mechanical Parameters of Swallowing Function

**DOI:** 10.1007/s13311-020-00996-2

**Published:** 2021-01-15

**Authors:** Sonja Suntrup-Krueger, Paul Muhle, Isabella Kampe, Paula Egidi, Tobias Ruck, Frank Lenze, Michael Jungheim, Richard Gminski, Bendix Labeit, Inga Claus, Tobias Warnecke, Joachim Gross, Rainer Dziewas

**Affiliations:** 1 Department of Neurology, University Hospital Muenster, Albert-Schweitzer-Campus 1 A, 48149 Muenster, Germany; 2 Institute for Biomagnetism and Biosignalanalysis, University Hospital Muenster, Malmedyweg 15, 48149 Muenster, Germany; 3 Pediatrics Department, St. Franziskus-Hospital Ahlen, Robert-Koch-Straße 55, 59227 Ahlen, Germany; 4 Department of Anesthesiology and Intensive Care Medicine, Clemenshospital Münster, Duesbergweg 124, 48153 Muenster, Germany; 5 Department of Medicine B for Gastroenterology and Hepatology, University Hospital Muenster, Albert-Schweitzer-Campus 1 A, 48149 Muenster, Germany; 6 Department of Phoniatrics and Pediatric Audiology, Hannover Medical School, Carl-Neuberg-Str. 1, 30625 Hannover, Germany; 7 Institute for Infection Prevention and Hospital Epidemiology, University Medical Center Freiburg, Faculty of Medicine, University of Freiburg, 79106 Freiburg, Germany

**Keywords:** Swallowing, Deglutition disorders, Dysphagia, Capsaicin, Substance P

## Abstract

Oropharyngeal dysphagia is prevalent in age-related neurological disorders presenting with impaired efficacy and safety of swallowing due to a loss of muscle force and sensory deficits. Stimulating the oropharynx with capsaicin that mediates Substance P release is an emerging pharmacological treatment option which needs further scientific evidence. Our aim was to comprehensively evaluate the effect of capsaicin on biochemical, neurophysiological, and biomechanical parameters of swallowing function. In a randomized study on healthy individuals, the impact of orally administered capsaicinoids at different dosages and application durations in comparison to non-carbonated water was evaluated. Time course and magnitude of salivary Substance P increase were monitored. Magnetoencephalography was used to detect cortical swallowing network alterations. Modifications in swallowing biomechanics were measured applying high-resolution pharyngeal manometry. Capsaicinoids at 10 μmol/L improved swallowing efficacy as seen by a significant increase of pharyngeal contractile integral and upper esophageal sphincter activation and relaxation times in manometry. Significant improvement of precision in a challenging swallow task accompanied by a reduction in swallowing-related submental electromyographic power was observed with capsaicinoids preconditioning at 10 μmol/L over 5 min, but not with continuous stimulation. The cortical activation pattern remained unchanged after any intervention. A significant increase of salivary Substance P was not detected with 10 μmol/L but with 50 μmol/L and lasted for 15 min after application. Capsaicinoids mediate dose-dependent Substance P release and positively alter swallowing biomechanics in healthy subjects. The results provide supportive evidence for the value of natural capsaicinoids to improve swallowing function.

**Supplementary Information:**

The online version contains supplementary material available at 10.1007/s13311-020-00996-2.

## Introduction

Swallowing is an essential neuromuscular function of the respiratory, oropharyngeal, and gastrointestinal structures to transport material from the mouth to the stomach while protecting the airway. Oropharyngeal dysphagia (OD), the disturbance of this complex sensorimotor function, is a prevalent comorbidity of various age-related neurological diseases and has recently been recognized as a geriatric syndrome [1]. It presents with impaired efficacy and safety of swallowing [2, 3] due to disturbed biomechanics caused by a loss of muscle force and function, reduction of tissue elasticity, saliva production, and gustatory function, impaired dental status, and reduced swallowing processing efficiency of the aging brain [4]. This is complemented by decreased oropharyngeal sensory perception due to a reduction of myelinated nerve fibers of the superior laryngeal nerve leading to delayed initiation of the swallow response [5]. OD causes severe complications that have a great impact on older peoples’ health and quality of life: malnutrition, dehydration, and aspiration pneumonia contribute to frailty and morbidity, lead to institutionalization, complicate hospital stay, foster readmissions, and increase mortality [2, 6].

In spite of the high prevalence and prognostic relevance of OD, therapeutic options are still limited. Conventional strategies mainly include compensatory maneuvers as well as texture-modified diet and thickening of fluids, which reduce the quality of life and are often limited by patient compliance resulting in insufficient fluid and caloric intake [7, 8]. Moreover, while these measures may improve swallowing safety, they do neither modify swallowing biomechanics nor do they promote swallowing recovery [9]. Accordingly, evidence for the effectiveness of these interventions is low [8, 10, 11].

Sensory afferent information plays a crucial role in the triggering and continuous adaptation of the motor swallow response [12, 13] within the distributed cortical and brainstem swallowing network [14, 15]. Therefore, a promising pharmacological strategy to treat OD is to enhance sensory input by stimulating the oropharynx with natural capsaicin. Capsaicinoids are acid amides with vanillylamide and C9–C11 branched-chain fatty acid [16]. They are found most abundantly in the genus Capsicum (family: Solanaceae). Capsaicin is a specific agonist to TRPV1 (transient receptor potential vanilloid subtype 1) [17], a polymodal sensory receptor expressed in the epithelial cells and sensory neurons of the oropharynx [18]. It mediates local release of Substance P (SP), a neuropeptide known to enhance the swallow and cough reflex [19] and to play an important role in the modulation of upper gastrointestinal motility [20, 21]. Dysphagia and aspiration pneumonia due to reduced airway protective mechanisms have been related to low SP concentration in elderly patients [22], after stroke [23], and in Parkinson’s disease [24]. Oral capsaicin supplementation increased salivary SP levels in a pilot study on geriatric OD [25] and changed swallow physiology in a beneficial manner [9, 26–29]. A neuromodulatory mechanism of action of capsaicin has been postulated but has rarely been investigated so far. Tomsen et al. found changes in the topography of the pharyngeal event-related potential after application of a different TRPV1 agonist (cinnamaldehyde + zinc) [30] and following repeated application of capsaicin over 10 days [31].

Despite these promising preliminary results, the use of capsaicin is still far from being implemented into clinical routine and needs further scientific evidence. The mechanism of action, the neurophysiological correlate of swallowing functional gains, and the temporal dynamics of the effect are incompletely understood. The aim of our study was therefore to comprehensively investigate the effect of oral application of a capsaicin-containing red pepper sauce suspension on the biomechanics and neurophysiology of swallowing. We took advantage of magnetoencephalography (MEG) as a functional brain imaging technique that is capable of detecting distributed cortical swallowing network activation, and high-resolution pharyngeal manometry (HRPM) as an objective, direct measurement of pharyngeal biomechanics. To gather further information on the feasibility of capsaicin treatment for dysphagia potential desensitization due to overstimulation was evaluated and the duration and intensity of the effect were assessed by monitoring salivary SP level over time.

## Methods

### Study Outline

**Fig. 1:**
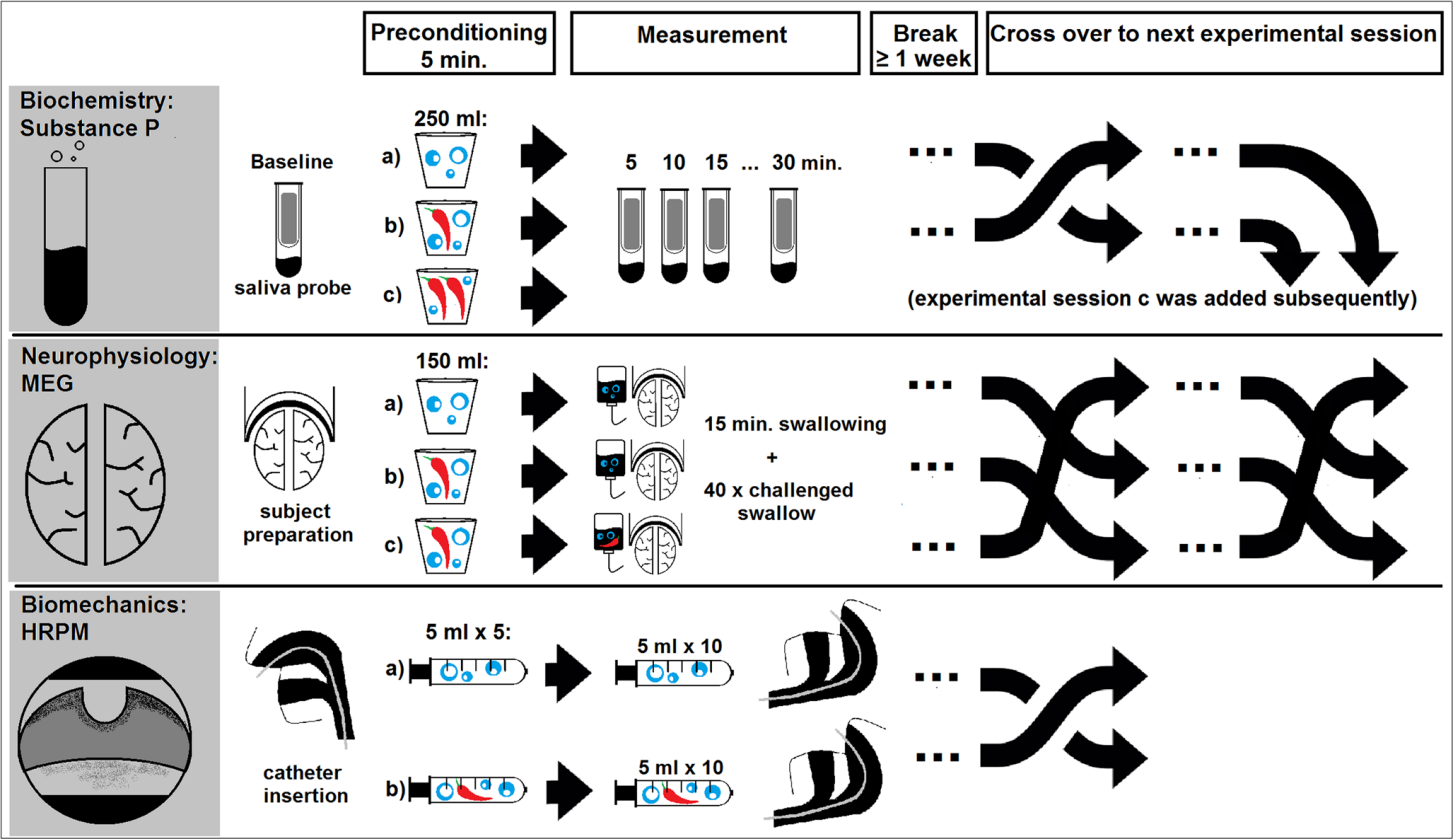
Study outline

This study included three separate experimental parts to evaluate the effects of oral capsaicinoids application on distinct aspects of swallowing function (Fig. 1): MEG was used to detect potential effects on the neurophysiology of swallowing. HRPM was applied to assess postulated changes in swallowing biomechanics and SP level in saliva was determined to evaluate underlying pharmacological properties.

All study parts were conducted independently from each other and designed in a placebo-controlled, randomized manner with exception of the high-dose capsaicinoids test series in the SP experiment, which was conducted thereafter (for details see below). Within each experimental part, measurement sessions were at least 1 week apart to avoid carryover effects. The sequence of the measurement sessions was created using computer-assisted randomization with Matlab (MathWorks Inc., USA).

### Subjects

Healthy adult volunteers were recruited for all parts of this study. Subjects had to be free of any neurologic, psychiatric, gastroenterologic, or ear-nose-throat disorder and did not take any medication affecting the neuromuscular, central nervous, or gastrointestinal system. They were instructed not to eat or drink for at least 4 h prior to participation in an experiment. The local ethics committee approved the protocol of the study. Informed consent was obtained from each subject after the nature of the study was explained in accordance with the principles of the declaration of Helsinki.

### Capsaicinoids

As a source of natural capsaicinoids, red pepper sauce (Tabasco, McIlhenny Co, Avery Island, USA) was used. Capsaicinoids concentrations in the sauce were determined by high-performance liquid chromatography using the AOAC official method 995.03 [32]. An overall capsaicinoid concentration of 196 μg/g was determined (capsaicin: 130 μg/g, dihydrocapsaicin: 60 μg/g, nordihydrocapsaicin 6 μg/g). For the experiments, final concentrations were obtained by dissolving the capsaicinoid-containig sauce in non-carbonated water as required for each experiment. A natural capsaicinoids sauce was used because it is an easily available alimentary product and has proved to be safe and effective in clinical pilot studies on elderly patients with swallowing dysfunction [9, 31].

### Substance P

Ten participants (30.6 ± 9.2 years, six male, four female) were recruited for this study part. The interventions in separate experimental sessions were oral application of a liquid which was either pure non-carbonated water or water supplemented with capsaicinoids (10 μmol/L). Participants were instructed to drink 250 mL of the respective liquid in small sips over 5 min. To assess the effect on SP concentration, saliva probes were taken before and 5, 10, 15, and 30 min after drinking the liquid. Saliva samples were collected using a salivette (Sarstedt, Nuembrecht, Germany) and centrifuged immediately at 4000 rpm for 5 min. Supernatants were stored in a deep freezer at − 20 °C until further analysis. SP levels were assessed by a competitive ELISA-type immunoassay parameter kit according to the manufacturer’s instructions (Substance P Immunoassay, catalog no. KGE007; R&D Systems, Minneapolis, USA).

As we could not detect the expected increase of SP level with capsaicinoids at 10 μmol/L (see results) another test series was afterwards conducted with a higher capsaicinoid dose of 50 μmol/L to further investigate a possible dose-dependent response.

### Magnetoencephalography

Magnetoencephalographic recordings of cortical brain activation during swallowing were performed in ten participants (25.7 ± 4.5 years, four male, six female) in three separate sessions.

Prior to any measurement, subjects had to drink 150 mL of a liquid in small sips over 5 min as preconditioning. During the following MEG measurements, a liquid was continuously infused into the oral cavity via a plastic tube attached to a reservoir bag with a flow rate of 10 mL/min to facilitate swallowing.

The three different study interventions were (a) preconditioning and continuous oral infusion with non-carbonated water, (b) preconditioning with water supplemented with capsaicinoids 10 μmol/L and continuous oral infusion of non-carbonated water during the measurement (“capsaicionid preconditioning only”), or (c) preconditioning and continuous infusion both with capsaicinoids-supplemented water at 10 μmol/L. The latter was performed to capture eventual desensitization effects due to overstimulation.

MEG data acquisition, preprocessing and statistical analysis was performed as established by our group in several previous studies [33–36]. The MEG system consisted of a 275-channel sensor array (Omega 275, CTF Systems Inc.). Onset and duration of swallows were identified by surface electromyographic (EMG) recording placed on submental muscles. Visual stimuli and feedback were delivered using Presentation software (NeuroBehavioral Systems, Inc., USA). Stimulus event markers and EMG were both coregistered with the MEG data for event-related analysis.

Subjects were measured in a sitting position looking at a screen approximately 80 cm in front of them. The tip of the infusion tube was placed in the mouth and fixed. During the first 15 min of the measurement, participants were instructed to swallow the infused liquid without external cueing. This was followed by a more demanding “challenged swallow” task as previously published [34]. In brief, participants had to swallow after a visual cue within a 150 ms target time interval. The task consisted of 40 trials and took roughly 10 min. After each swallow visual feedback (icons for “hit,” “early,” “late,” or “error” in case no swallow was identified) was given to the subject according to EMG swallowing activity in relation to the stimulus event markers defining the target time interval.

Subsequent MEG data processing and statistical analysis were carried out with custom-made Matlab scripts based on FieldTrip (http://www.ru.nl/fcdonders/fieldtrip) [37] as previously described in detail elsewhere [34–36]. In brief, MEG data were filtered within theta (4–8 Hz), alpha (8–13 Hz), beta (13–30 Hz), low gamma, (30–60 Hz), and high gamma (60–80 Hz) frequency bands. Source localization of each subject’s swallowing-associated cortical activation was done by applying a beamformer as this method is capable of analyzing induced brain activity such as the event-related desynchronization (ERD) of cortical rhythms which occurs during sensorimotor tasks [38]. Each subjects’ volumetric source estimates were spatially normalized to a template MNI brain (T1, Montreal Neurological Institute, Canada) using SPM8 (http://www.fil.ion.ucl.ac.uk/spm). Grandaverages of normalized and realigned source activation maps were computed across subjects. To identify significant (*p* < 0.05) brain activation changes induced by capsaicinoids, source grandaverages of the “capsaicinoids preconditioning,” and the “continuous capsaicinoids” conditions were both compared to the water condition applying a cluster-based nonparametric randomization approach built into FieldTrip. Significance values were Bonferroni-corrected. As tongue movement during swallowing is known to produce a strong source reconstruction artifact at the base of the brain and the inferior temporal lobes, statistical analysis was restricted to a region of interest consisting of the frontal and parietal lobe, and the insula, thereby including those brain regions that have consistently been found to be associated with swallowing in prior neuroimaging studies and can reliably be localized with MEG [36].

Besides its influence on cortical brain activation, effects of the intervention on performance in the challenged swallow task, i.e. the proportion of hits, and on swallowing muscle EMG parameters, i.e. power (root mean square (RMS) value) and maximum peak-to-peak amplitude, were assessed. Head movement during the measurement and swallow count were documented to ensure similar performance.

### High-Resolution Pharyngeal Manometry

Twenty subjects (29.0 ± 6.4 years, twelve male, eight female) participated in the HRPM as an objective, direct measurement of pharyngeal biomechanics. As study intervention, the liquid that was given as preconditioning and to facilitate swallowing during HRPM was either pure non-carbonated water or water supplemented with capsaicinoids (10 μmol/L).

For examination, the ManoScan™ workstation and ManoScan™ ESO Z catheter (Medtronic, Sunnyvale, CA, USA) containing 36 circumferential pressure sensors spaced 1 cm apart and 18 impedance channels at a 4.2-mm diameter were used. Prior to every measurement, the catheter was calibrated according to the manufacturer’s specifications. Then, it was inserted transnasally and introduced into the esophagus while the subject performed sequential swallows. Correct placement of the catheter was confirmed by mapping of the typical pharyngeal pressure topography plot which had to span the velopharynx and the upper esophageal sphincter (UES). Afterwards, participants had at least 5 min time to get accustomed to the catheter. As preconditioning prior to the measurement, they were given capsaicinoid-supplemented water or tap water, respectively, at a rate of 5 mL/min for 5 min. The examination was performed in a supine position starting with a 30 s baseline pressure recording followed by a series of ten cued 5-mL swallows of liquid at room temperature administered via syringe with one swallow every 30 s.

Data analysis was performed using ManoView™ ESO 3.3 software (Medtronic, Sunnyvale, CA, USA). To examine the influence of capsaicinoids on the dynamic swallowing behavior of the velopharynx, base of tongue, and upper esophageal sphincter (UES) the following parameters were assessed, which have been described in detail elsewhere [39–43]: lumen contractile peak pressures at the velum and tongue base, duration of velopharyngeal and tongue base contraction (from initial pressure increase until return to baseline), UES maximum occlusive pressures pre- and postrelaxation, UES mean basal pressure (within 5 s prior to a swallow), UES relaxation nadir pressure, UES activation time (interval between maximum pressures pre- and postrelaxation), and UES relaxation time (starting at a 10% sphincter pressure drop and ending with return to the same value when the pharyngeal contraction wave arrives) [39]. In addition, the Pharyngeal Contractile Integral (PhCI), which defines pressure over space and time (pharyngeal pressure amplitude × length of the pharynx × duration of contraction) in mmHg cm s was used as a recommended measure of the “vigor” of the entire pharyngeal swallowing [42].

### Statistical Analysis

Apart from MEG data analysis statistical computations were carried out using SPSS Statistics 25.0 (IBM Corporation, Armonk, USA). Prior to any comparison normality testing was performed using the Kolmogorov-Smirnov test. In case of a normal distribution, intervention effects (water *vs* continuous capsaicinoids *vs* capsaicinoids preconditioning) on MEG task performance were assessed by repeated measures ANOVA, otherwise Friedman test was applied as a nonparametric counterpart. For every ANOVA Mauchly’s test evaluated the sphericity assumption and Greenhouse–Geisser correction was applied in case of violation. In case the ANOVA turned significant, intervention effects were analyzed post hoc applying paired sample *t* tests with Bonferroni correction. In case Friedman’s test turned significant, a post hoc Wilcoxon test was used with multiple comparison correction, respectively.

To account for the high interindividual variability and for day-related intraindividual variations, saliva SP values were normalized through division by the respective baseline values. To evaluate the effect of water *vs* low-dose capsaicinoids, or high-dose capsaicinoids on saliva SP levels over time, repeated measures ANOVA including factors (intervention x time) were performed as described above.

For comparison of HRPM parameters between the water *vs* capsaicinoids condition a paired sample *t* test was completed, or the Wilcoxon test was used if data failed normality testing. Significance was accepted as *p* value ≤ 0.05.

## Results

### Substance P

**Fig. 2:**
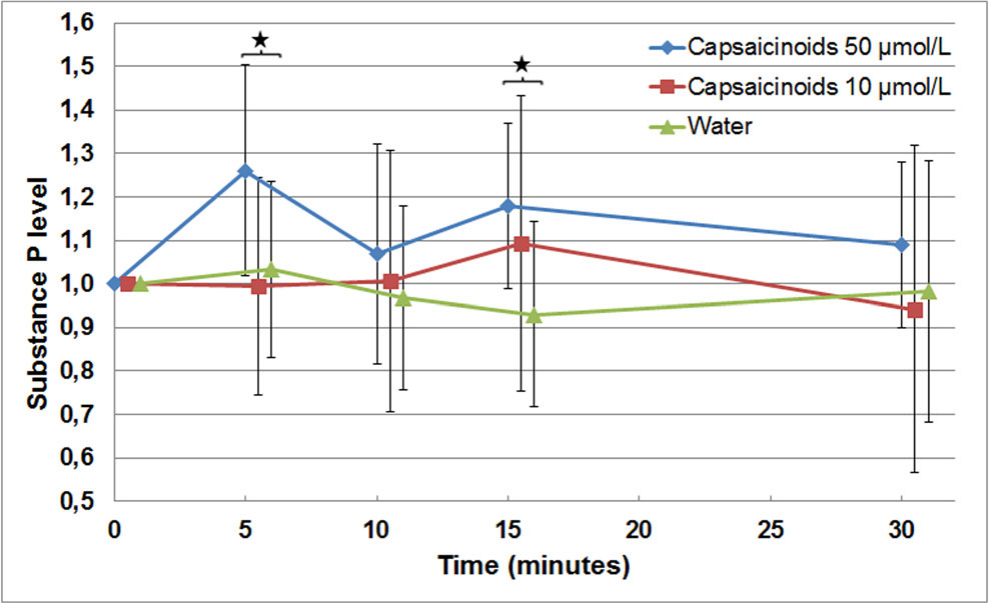
Time course of saliva Substance P levels according to the study intervention (baseline-normalized mean values; vertical bars indicate standard deviations; the asterisk indicates statistical significance)

The time course of baseline-normalized SP concentration in saliva in the three test series is shown in Fig. 2. No significant changes in SP levels were induced in the initial test series with capsaicinoids at 10 μmol/L. At a higher concentration of 50 μmol/L, a significant intervention effect was found [F(1,10) = 6.153, *p* = 0.033]. Post hoc comparison demonstrated relevant SP increase with high-dose capsaicinoids compared to water at five (26.3%, *p* = 0.025) and 15 min (17.7%, *p* = 0.015).

### High-Resolution Pharyngeal Manometry

There were no complaints of any relevant discomfort related to HRPM and all measurements were successfully completed. HRPM results are summarized in Table 1. Capsaicinoids significantly prolonged UES activation time by 60 ms and UES relaxation time by 30 ms. Moreover, capsaicinoid administration induced a more vigorous pharyngeal swallow: the Pharyngeal Contractile Integral was significantly increased by 33.1 mmHg cm s in mean. As the length of the pharynx is assumed to be constant in each individuum, the change is caused by an increase of the pharyngeal pressure amplitude and/or contraction duration.

**Table 1:** High-resolution pharyngeal manometry results

	Pure water	Capsaicinoids	*p* value
Max. velopharyngeal closing pressure, mmHg	148.3 ± 39.3	146.6 ± 37.8	0.850
Duration of velopharyngeal contraction, s	0.71 ± 0.08	0.74 ± 0.07	0.079
Max. tongue base pressure, mmHg	242.0 ± 104.7	263.9 ± 132.2	0.279
Duration of tongue base contraction, s	0.70 ± 0.08	0.72 ± 0.09	0.138
UES^†^ max. pressures predeglutition, mmHg	100.6 ± 42.3	109.7 ± 44.3	0.298
UES^†^ max. pressures postdeglutition, mmHg	240.3 ± 76.4	253.7 ± 89.9	0.189
UES^†^ resting pressure, mmHg	30.4 ± 11.4	32.9 ± 10.2	0.224
UES^†^ relaxation nadir pressure, mmHg	− 8.3 ± 4.6	− 9.8 ± 5.4	0.090
UES^†^ relaxation time, s	0.69 ± 0.07	0.72 ± 0.06	0.028*
UES^†^ activation time, s	0.87 ± 0.11	0.93 ± 0.07	0.004*
Pharyngeal contractile integral, mmHg cm s	252.6 ± 148.6	285.7 ± 147.9	0.019*

Data are given as mean ± standard deviation

*Indicates statistical significance

^†^Upper esophageal sphincter

### Magnetoencephalography

Comparable subject performance in the MEG over the different sessions was confirmed by similar swallow count and head movement (Table 2). Consistent EMG recording technique was confirmed by similar EMG amplitude, indicating similar skin resistance and electrode positioning in all sessions (see also Table 2).

**Table 2:** MEG task performance data

	Pure water	Capsaicinoid preconditioning	Continuous capsaicinoids	*p* value
Head movement (mm), mean ± SD				
Volitional swallowing	0.35 ± 0.23	0.46 ± 0.39	0.37 ± 0.21	1.000
Challenged swallow task	0.22 ± 0.12	0.23 ± 0.14	0.35 ± 0.34	0.905
Swallow count, *n* ± SD				
Volitional swallowing	72 ± 18	75 ± 30	71 ± 13	0.905
Hits in challenged swallow, % ± SD	43.2 ± 11.5	67.1 ± 15.9	46.2 ± 9.1	< 0.0005*
EMG^†^ power (μV), mean ± SD				
Volitional swallowing	161.9 ± 178.9	55.0 ± 15.3	107.1 ± 49.6	0.002*
Challenged swallow task	95.8 ± 41.2	63.2 ± 12.3	122.4 ± 74.9	0.014*
EMG^†^ amplitude (μV), mean ± SD				
Volitional swallowing	659.4 ± 440.5	463.4 ± 140.1	597.8 ± 197.4	0.273
Challenged swallow task	519.2 ± 145.5	548.2 ± 150.1	604.1 ± 322.6	0.584

*Indicates statistical significance in analysis of variance (ANOVA) or Friedman’s test

^†^Electromyographic

**Fig. 3:**
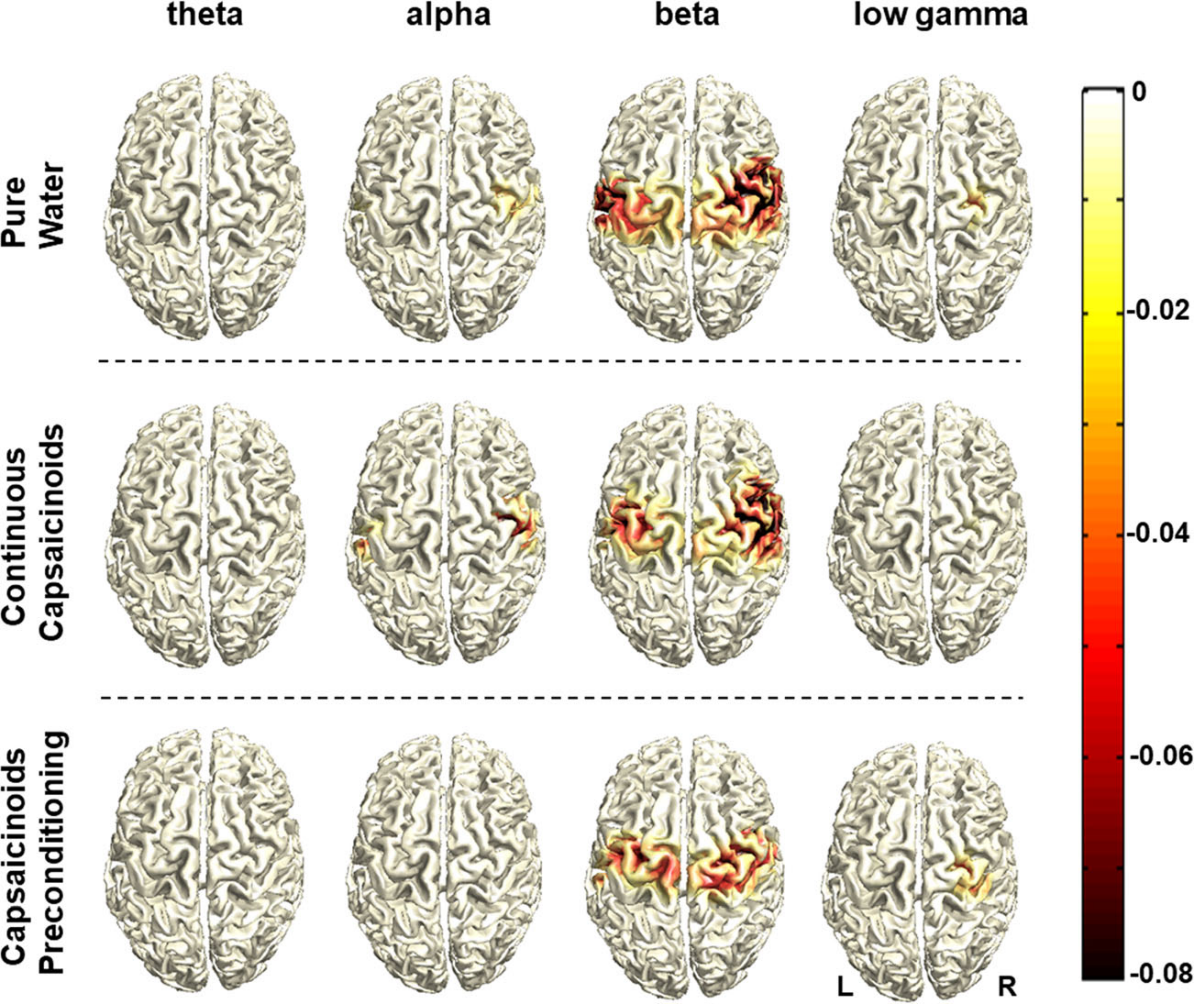
Source distribution of group-average task-related cortical activation during voluntary swallowing. Data are displayed for all three conditions and per frequency band. The color bar with negative values indicates event-related desynchronization of oscillatory activity relative to the resting stage

**Fig. 4:**
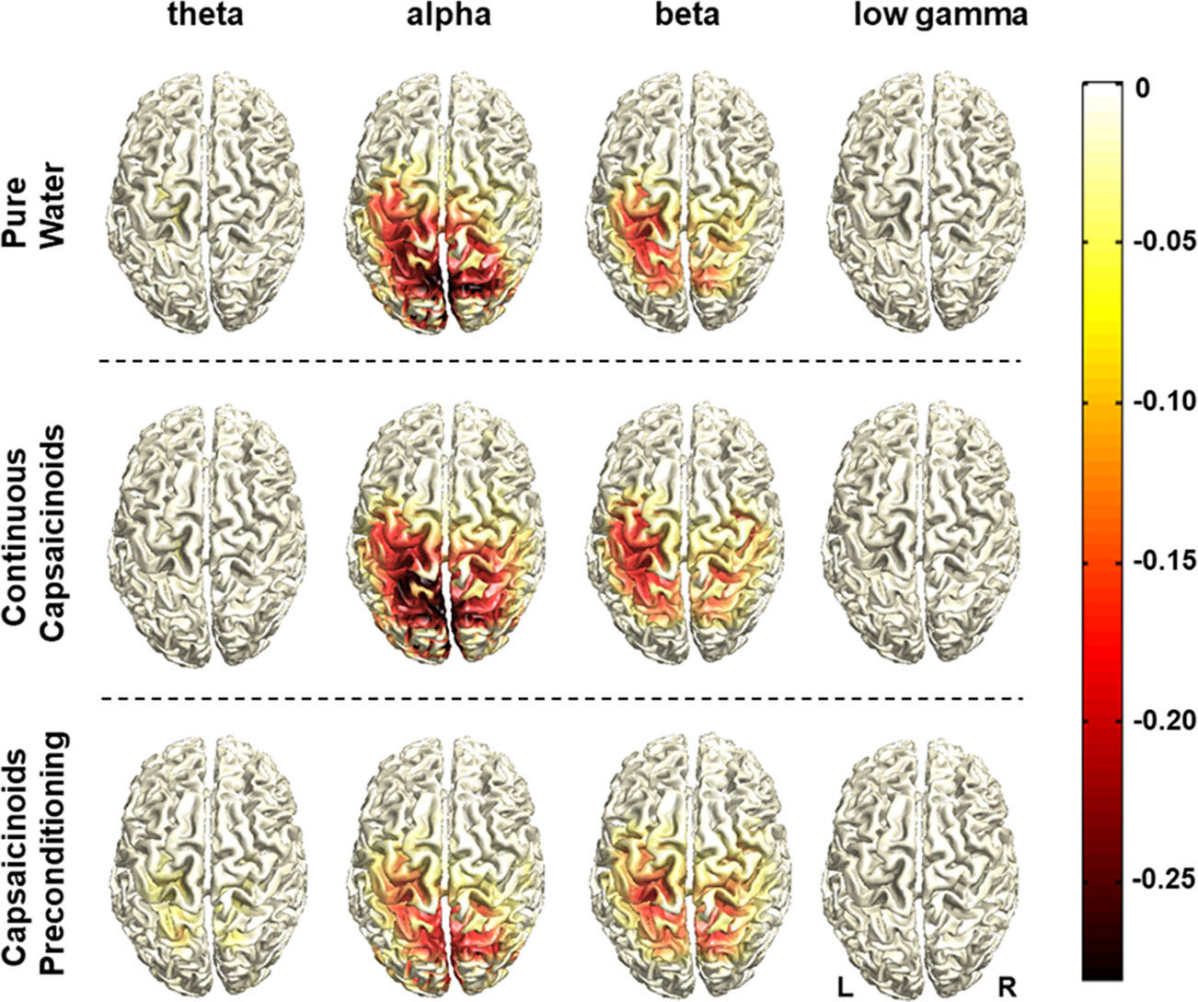
Source distribution of group-average task-related cortical activation during the challenged swallow. Data are displayed for all three conditions and per frequency band. The color bar with negative values indicates event-related desynchronization of oscillatory activity relative to the resting stage

Swallowing resulted in ERD of oscillatory brain activity visible from the theta to the low gamma range with no task-related activation changes in high gamma. Voluntary swallowing activation centered in the “motor” beta frequency band and was localized in the bilateral pericentral cortex corresponding to primary and secondary sensorimotor areas (Fig. 3). With the visually cued challenged swallow task, the parieto-occipital visual cortex was also found to be activated in the “visual” alpha band and less strongly in the beta range (Fig. 4). Significant effects of the capsaicinoids application on the cortical activation pattern compared to pure water were neither found in the “capsaicinoids preconditioning” nor in the “continuous capsaicinoids” condition in any swallowing task. However, swallowing performance in the challenged task was significantly improved by capsaicinoids preconditioning (Table 2). Percentage of hits was increased by more than 20% (post hoc *t* tests, Bonferroni-corrected: capsaicinoid preconditioning *vs* pure water: *p* < 0.0015, capsaicinoid preconditioning *vs* continuous capsaicinoids: *p* < 0.0015; pure water *vs* continuous capsaicinoids: *p* = 0.681). Moreover, EMG power (Table 2) was significantly lower with capsaicinoid preconditioning than in the other two conditions during volitional swallowing (post hoc Wilcoxon test, Bonferroni-corrected: capsaicinoids preconditioning *vs* continuous capsaicinoids: *p* = 0.015; capsaicinoids preconditioning *vs* pure water: *p* = 0.021; pure water *vs* continuous capsaicinoids: *p* = 1.0), and at least lower compared to continuous capsaicinoids in the challenged swallow task (post hoc Wilcoxon test, Bonferroni-corrected: capsaicinoids preconditioning *vs* continuous capsaicinoids: *p* = 0.015; capsaicinoids preconditioning *vs* pure water: *p* = 0.141; pure water *vs* continuous capsaicinoids: *p* = 0.999).

## Discussion

The administration of capsaicin-containing red pepper sauce suspension to healthy individuals significantly modified swallowing as seen by greater vigor of the pharyngeal swallow and longer UES relaxation duration in HRPM and by increased precision in the MEG challenged swallow task. This effect was not found to be related to alterations in cortical swallowing processing but a dose-dependent rise of saliva SP level for at least 15 min after application was observed as an indicator of effective sensory afferent stimulation. No adverse reactions occurred to capsaicinoids application.

### Pharmacology

As far as we know there is only one recent study to show salivary SP increase after oral capsaicin administration [25]. While a dose-dependent acceleration of swallow reflex triggering [44, 45] has been demonstrated and a dose-dependency of salivary SP increase could therefore be postulated our study is the first to prove this concept: although we found significant effects on swallowing function with 10 μmol/L, a relevant increase of SP could only be measured at a 5-fold higher dose. Slight increases in SP level with 10 μmol/L may have been missed due to dilution effects in saliva. The optimal concentration and pharyngeal mucosa contact time of capsaicin are still unknown. Dosage forms in previous studies vary from a liquid suspension [5] via tablets [26] or thin films [25] to natural capsaicin-containing foods [27]. As a consequence final concentrations when dissolving in the mouth are hard to infer rendering profound conclusions difficult. While some authors found significant effects on swallowing kinematics at low doses up to 1 μmol/L [26, 44, 45] others preferred higher concentrations around 150 μmol/L, also with good results [9, 29, 46]. However, the pungency at this concentration was reported to be a problem [9, 31]. Moreover, high concentrations or chronic application have been shown to desensitize the release of SP [17]. In vitro desensitization effects in sensory neurons have even been observed with concentrations as low as 10 μmol/L [47], whereas clinical effects in patients remained stable over the treatment period with 10 μmol/L [5] and 150 μmol/L [9].

Based on the available evidence we initially chose a medium concentration of 10 μmol/L which we assumed to be effective and at the same time acceptable even if administered continuously during a measurement. Capsaicinoids preconditioning time was 5 min in all three study parts because this time span proved to be sufficiently long to induce a sensitization [9, 20, 26, 46] but short enough to prevent desensitization. According to our SP results, we indeed did not observe any signs of desensitization. There was no early SP level peak with consecutive drop due to emptying of TRPV1 receptors at 10 μmol/L. SP increase was rather detected with a five-fold higher concentration and was still above baseline at 30 min. Our study was also the first to directly measure the time course of salivary SP increase after stimulation. Results are within the range reported by Grossi et al. [48] who detected a return to baseline in the manometric esophageal motility pattern as an indirect parameter of SP release 15 to 25 min after intraluminal administration of capsaicin. In terms of clinical applicability, the increase of SP seems to last long enough to complete a meal.

### Biomechanics

It is well-established with esophageal manometry that capsaicin exerts a prokinetic effect on esophageal motility by increasing the contractile amplitude, duration, and velocity of esophageal body peristalsis [20, 21, 48]. In analogy, we applied pharyngeal manometry, a relatively new manometric application [40], to assess the effect of capsaicin on oropharyngeal swallowing biomechanics. The pressure forces that are measurable by HRPM are influenced by the strength and sequencing of the swallowing muscles which are modulated by peripheral sensory and cortical inputs to the swallowing central pattern generator in the brainstem [40]. As capsaicin likely stimulates these afferent sensory nerves in the pharyngeal plexus [26] we postulated an increase in pharyngeal contractility. Indeed, the baseline value of the PhCI was within the normal range reported in healthy individuals [49] and increased significantly after capsaicinoids application.

Whereas a more vigorous contractility in the pharynx was observed in healthy older individuals and is regarded a physiological compensatory response for other age-related changes in swallowing function [49], geriatric OD or “presbyphagia” typically presents with weak pharyngeal muscular contraction. The PhCI, representing the “vigor” of the swallow, was therefore established as a useful indicator of pharyngeal swallowing impairment [43, 50]. Reduced UES relaxation duration is another hallmark of sarcopenic OD [50, 51]. Capsaicin was able to positively influence both of these parameters in our study indicating that capsaicin treatment may be especially useful to improve swallowing efficacy in elderly people. In line with that, TRPV1 stimulation with capsaicinoids reduced pharyngeal residue by 80% in a previous videofluoroscopy study [45].

The findings that were most consistently reported from EMG and videofluoroscopy studies after capsaicinoid administration are a shortening of swallow response time and the time to laryngeal vestibule closure [9, 26, 27, 44, 45, 52]. Unfortunately, these measures cannot easily be investigated with HRPM because they have no salient manometric correlate [53]. However, better performance in the MEG challenged swallow task, for which precise timing of laryngeal elevation was of special importance [34], is in line with videofluoroscopic results.

It might be surprising that submental EMG power decreased in the MEG volitional swallowing task after capsaicinoids preconditioning while—according to the HRPM results—one could have expected an increase in muscle contractility in all swallow-relevant muscle groups. Hoffman et al. [53] observed disordered hyoid excursion, which is generated by submental muscles, despite regular pharyngoesophageal segment opening in a study on OD, indicating that the two muscle groups work independently and may also be modulated differently. It may be that capsaicinoids preconditioning led to a more efficient pharyngoesophageal passage as reflected in HRPM results requiring less submental muscle effort to perform precise swallowing in the MEG. Contrary, continuous capsaicinoids application over 30 min caused neither changes in the EMG nor in the challenged swallow task indicating potential desensitization due to overstimulation.

### Neurophysiology

The swallowing-associated cortical activation pattern was consistent with former MEG studies in healthy subjects: swallowing is coordinated bilaterally in a distributed yet focal network involving among others the primary and secondary sensorimotor areas, the insula, and anterior cingulate [34, 35, 54, 55].

Pharyngeal electrical stimulation (PES), a neuromodulation technique which also operates via local pharyngeal SP release [56, 57] has been shown to induce reorganization of neural connections within the cortical swallowing network thereby improving swallowing function [35, 58, 59]. In analogy, SP-releasing pharmacological TRPV1 agonists have been postulated to increase the sensory input to the brainstem and cortical areas, facilitating deglutition. With the experimental setup applied here, however, we were not able to prove this concept. There are several reasons why we may have missed underlying neurophysiological changes despite clear effects on swallowing function in the MEG challenged swallow task: First, the effect size of a single application in healthy subjects is probably small and might not be detected with our relatively low number of participants. Second, MEG is mostly sensitive to cortical neuronal activity. Changes in subcortical and brainstem circuits, where the swallowing pattern generator is located, may have been missed. Tomsen et al. [31] observed increased cortical event-related potentials in the cingulate gyrus and the medial frontal gyrus, two areas involved in stimulus perception and swallowing preparation, after longer-term capsaicinoids stimulation over 10 days, but not after one-time intervention. The most plausible explanation for the lack of cortical changes after single application is a potential dual effect of capsaicin on swallowing function [27, 29]: On the one hand, it has a direct short-term facilitatory effect on sensory neurons that immediately improves afferent input from the pharynx, promoting the pharyngeal phase of swallowing. Secondly, it probably induces an indirect neuromodulatory long-term effect through repetitive stimulation to the cortex. This would explain beneficial behavioral effects after a single stimulation episode but no immediate effects in brain imaging, only after repetitive stimulation [31].

### Clinical Implications

Given that the pathophysiology of OD in seniors is substantially based on impaired swallowing biomechanics and decreased oropharyngeal sensitivity [46], the dual, motor and sensory effect of capsaicinoids demonstrated in our study makes it a suitable treatment option in geriatric care. A concentration of 10 μmol/L was well-tolerated by our participants and seems to be safe and acceptable also for older patients with oropharyngeal dysphagia [5, 31]. Supplementing the food with capsaicin would be a widely available, easy to administer, and low-cost strategy to improve swallowing function in elderly persons with presbyphagia who are still on an oral diet. Based on our results and previous clinical studies [20, 26, 46] it seems enough to administer capsaicin over a few minutes prior to a meal to get an effect that lasts long enough to complete this meal. Daily stimulation is probably beneficial to build up and maintain a neuromodulatory long-term effect [27, 29, 31].

### Limitations

This study was designed as a proof-of-principle investigation and therefore conducted in a low number of healthy participants. Future studies should aim to replicate the findings in the population of elderly subjects. Further clinical trials are also needed to determine the optimal way of application, treatment interval, and minimal effective dose of capsaicin to avoid desensitization and reduce pungency. Given the capsaicin effect on swallowing biomechanics at a concentration of 10 μmol/l in the absence of a detectable substance P increase, we cannot rule out that other stimulus effects of capsaicin may (additionally) have impacted on the behavioral results.

The HRPM method applied here is relatively new and despite a body of published evidence and a critical mass of investigators in the field, there is a lack of consensus surrounding what biomechanical phenomena to measure [41]. We decided on evaluating parameters that are particularly relevant in the context of geriatric OD. The more conventional method of videofluoroscopy was not applicable in our healthy volunteers because of the exposure to X-ray. Fiberoptic endoscopic evaluation of swallowing as an alternative method was not chosen because it gives semiquantitative results only and was suspected to potentially miss slight intervention effects that occur when investigating healthy participants.

## Conclusion

In summary, our results in healthy subjects provide supportive evidence for the value of natural capsaicinoids to treat OD. Findings may contribute towards moving on dysphagia therapy from sole compensation to true recovery of swallowing function.

## Supplementary Information

ESM 1 (PDF 384 kb)

## References

[1] Baijens LW, Clave P, Cras P (2016). European Society for Swallowing Disorders - European Union Geriatric Medicine Society white paper: oropharyngeal dysphagia as a geriatric syndrome. Clin Interv Aging..

[2] Clave P, Rofes L, Carrion S (2012). Pathophysiology, relevance and natural history of oropharyngeal dysphagia among older people. Nestle Nutr Inst Workshop Ser..

[3] Muhle P, Wirth R, Glahn J (2015). Age-related changes in swallowing. Physiology and pathophysiology. Nervenarzt..

[4] Teismann IK, Steinstraeter O, Schwindt W (2010). Age-related changes in cortical swallowing processing. Neurobiol Aging..

[5] Ortega O, Rofes L, Martin A (2016). A Comparative Study Between Two Sensory Stimulation Strategies After Two Weeks Treatment on Older Patients with Oropharyngeal Dysphagia. Dysphagia..

[6] Humbert IA, Robbins J (2008). Dysphagia in the elderly. Phys Med Rehabil Clin N Am..

[7] Burkhead LM, Sapienza CM, Rosenbek JC (2007). Strength-training exercise in dysphagia rehabilitation: principles, procedures, and directions for future research. Dysphagia..

[8] Geeganage C, Beavan J, Ellender S (2012). Interventions for dysphagia and nutritional support in acute and subacute stroke. Cochrane Database Syst Rev..

[9] Rofes L, Arreola V, Martin A (2013). Natural capsaicinoids improve swallow response in older patients with oropharyngeal dysphagia. Gut..

[10] Speyer R, Baijens L, Heijnen M (2010). Effects of therapy in oropharyngeal dysphagia by speech and language therapists: a systematic review. Dysphagia..

[11] Suntrup-Krueger S, Minnerup J, Muhle P (2018). The Effect of Improved Dysphagia Care on Outcome in Patients with Acute Stroke: Trends from 8-Year Data of a Large Stroke Register. Cerebrovasc Dis..

[12] Teismann IK, Steinstraeter O, Stoeckigt K (2007). Functional oropharyngeal sensory disruption interferes with the cortical control of swallowing. BMC Neurosci..

[13] Muhle P, Suntrup-Krueger S, Dziewas R (2018). Pharyngeal dysphagia due to Varicella zoster virus meningoradiculitis and full recovery: Case report and endoscopic findings. SAGE Open Med Case Rep.

[14] Jean A (2001). Brain stem control of swallowing: neuronal network and cellular mechanisms. Physiol Rev..

[15] Michou E, Hamdy S (2009). Cortical input in control of swallowing. Curr Opin Otolaryngol Head Neck Surg..

[16] Huang X-F, Xue J-Y, Jiang A-Q (2013). Capsaicin and its Analogues: Structure-Activity relationship study. Curr Med Chem.

[17] Caterina MJ, Schumacher MA, Tominaga M (1997). The capsaicin receptor: a heat-activated ion channel in the pain pathway. Nature..

[18] Alvarez-Berdugo D, Rofes L, Farre R (2016). Localization and expression of TRPV1 and TRPA1 in the human oropharynx and larynx. Neurogastroenterol Motil..

[19] Jin Y, Sekizawa K, Fukushima T (1994). Capsaicin desensitization inhibits swallowing reflex in guinea pigs. Am J Respir Crit Care Med..

[20] Chen CL, Liu TT, Yi CH (2010). Effects of capsaicin-containing red pepper sauce suspension on esophageal secondary peristalsis in humans. Neurogastroenterol Motil..

[21] Gonzalez R, Dunkel R, Koletzko B (1998). Effect of capsaicin-containing red pepper sauce suspension on upper gastrointestinal motility in healthy volunteers. Dig Dis Sci..

[22] Nakagawa T, Ohrui T, Sekizawa K (1995). Sputum substance P in aspiration pneumonia. Lancet..

[23] Arai T, Yoshimi N, Fujiwara H (2003). Serum substance P concentrations and silent aspiration in elderly patients with stroke. Neurology..

[24] Schroder JB, Marian T, Claus I (2019). Substance P Saliva Reduction Predicts Pharyngeal Dysphagia in Parkinson's Disease. Front Neurol..

[25] Nakato R, Manabe N, Shimizu S (2017). Effects of Capsaicin on Older Patients with Oropharyngeal Dysphagia: A Double-Blind, Placebo-Controlled, Crossover Study. Digestion.

[26] Ebihara T, Takahashi H, Ebihara S (2005). Capsaicin troche for swallowing dysfunction in older people. J Am Geriatr Soc..

[27] Shin S, Shutoh N, Tonai M (2016). The Effect of Capsaicin-Containing Food on the Swallowing Response. Dysphagia..

[28] Ebihara S, Kohzuki M, Sumi Y (2011). Sensory stimulation to improve swallowing reflex and prevent aspiration pneumonia in elderly dysphagic people. J Pharmacol Sci..

[29] Wang Z, Wu L, Fang Q (2019). Effects of capsaicin on swallowing function in stroke patients with dysphagia: A randomized controlled trial. J Stroke Cerebrovasc Dis..

[30] Tomsen N, Alvarez-Berdugo D, Rofes L (2020). A randomized clinical trial on the acute therapeutic effect of TRPA1 and TRPM8 agonists in patients with oropharyngeal dysphagia. Neurogastroenterol Motil..

[31] Tomsen N, Ortega O, Rofes L (2019). Acute and subacute effects of oropharyngeal sensory stimulation with TRPV1 agonists in older patients with oropharyngeal dysphagia: a biomechanical and neurophysiological randomized pilot study. Therap Adv Gastroenterol..

[32] Association of Official Analytical Chemists (AOAC). Official Method 995.03: Capsaicinoids in Capsicums and their Extractives - Liquid Chromatographic Method. AOAC Official Methods of Analysis 2000, 17th Edition, 43:14-16.

[33] Suntrup S, Teismann I, Bejer J (2013). Evidence for adaptive cortical changes in swallowing in Parkinson's disease. Brain..

[34] Suntrup S, Teismann I, Wollbrink A (2013). Magnetoencephalographic evidence for the modulation of cortical swallowing processing by transcranial direct current stimulation. Neuroimage..

[35] Suntrup S, Teismann I, Wollbrink A (2015). Pharyngeal electrical stimulation can modulate swallowing in cortical processing and behavior — Magnetoencephalographic evidence. Neuroimage..

[36] Suntrup-Krueger S, Ringmaier C, Muhle P (2018). Randomized trial of transcranial direct current stimulation for poststroke dysphagia. Ann Neurol..

[37] Oostenveld R, Fries P, Maris E (2011). FieldTrip: Open source software for advanced analysis of MEG, EEG, and invasive electrophysiological data. Comput Intell Neurosci..

[38] Pfurtscheller G (2001). Functional brain imaging based on ERD/ERS. Vision Res..

[39] Jungheim M, Schubert C, Miller S (2015). Normative Data of Pharyngeal and Upper Esophageal Sphincter High Resolution Manometry. Laryngorhinootologie..

[40] Jungheim M, Ptok M (2018). High-resolution manometry of pharyngeal swallowing dynamics. HNO..

[41] Omari T, Schar M (2018). High-resolution manometry: what about the pharynx?. Curr Opin Otolaryngol Head Neck Surg..

[42] Omari TI, Ciucci M, Gozdzikowska K (2020). High-Resolution Pharyngeal Manometry and Impedance: Protocols and Metrics-Recommendations of a High-Resolution Pharyngeal Manometry International Working Group. Dysphagia..

[43] O'Rourke A, Humphries K, Lazar A, et al. The pharyngeal contractile integral is a useful indicator of pharyngeal swallowing impairment. Neurogastroenterol Motil. 2017; 29:10.1111/nmo.13144. 10.1111/nmo.13144 PMC5690888 28699250

[44] Ebihara T, Sekizawa K, Nakazawa H (1993). Capsaicin and swallowing reflex. Lancet..

[45] Yamasaki M, Ebihara S, Ebihara T (2010). Effects of capsiate on the triggering of the swallowing reflex in elderly patients with aspiration pneumonia. Geriatr Gerontol Int..

[46] Alvarez-Berdugo D, Rofes L, Arreola V, et al. A comparative study on the therapeutic effect of TRPV1, TRPA1, and TRPM8 agonists on swallowing dysfunction associated with aging and neurological diseases. Neurogastroenterol Motil. 2018;30:10.1111/nmo.13185. 10.1111/nmo.13185 28799699

[47] Kiraly E, Jancso G, Hajos M (1991). Possible morphological correlates of capsaicin desensitization. Brain Res..

[48] Grossi L, Cappello G, Marzio L (2006). Effect of an acute intraluminal administration of capsaicin on oesophageal motor pattern in GORD patients with ineffective oesophageal motility. Neurogastroenterol Motil..

[49] Nativ-Zeltzer N, Logemann JA, Zecker SG (2016). Pressure topography metrics for high-resolution pharyngeal-esophageal manofluorography-a normative study of younger and older adults. Neurogastroenterol Motil..

[50] Kunieda K, Fujishima I, Wakabayashi H, et al. Relationship Between Tongue Pressure and Pharyngeal Function Assessed Using High-Resolution Manometry in Older Dysphagia Patients with Sarcopenia: A Pilot Study. Dysphagia 2020; 10.1007/s00455-020-10095-1. Online ahead of print. 10.1007/s00455-020-10095-1 32140906

[51] Park CH, Kim DK, Lee YT (2017). Quantitative Analysis of Swallowing Function Between Dysphagia Patients and Healthy Subjects Using High-Resolution Manometry. Ann Rehabil Med..

[52] Rofes L, Arreola V, Martin A (2014). Effect of oral piperine on the swallow response of patients with oropharyngeal dysphagia. J Gastroenterol..

[53] Hoffman MR, Jones CA, Geng Z (2013). Classification of high-resolution manometry data according to videofluoroscopic parameters using pattern recognition. Otolaryngol Head Neck Surg..

[54] Furlong PL, Hobson AR, Aziz Q (2004). Dissociating the spatio-temporal characteristics of cortical neuronal activity associated with human volitional swallowing in the healthy adult brain. Neuroimage..

[55] Dziewas R, Soros P, Ishii R (2003). Neuroimaging evidence for cortical involvement in the preparation and in the act of swallowing. Neuroimage..

[56] Suntrup-Krueger S, Bittner S, Recker S (2016). Electrical pharyngeal stimulation increases substance P level in saliva. Neurogastroenterol Motil..

[57] Muhle P, Suntrup-Krueger S, Bittner S (2017). Increase of Substance P Concentration in Saliva after Pharyngeal Electrical Stimulation in Severely Dysphagic Stroke Patients - an Indicator of Decannulation Success?. Neurosignals..

[58] Suntrup S, Marian T, Schroder JB (2015). Electrical pharyngeal stimulation for dysphagia treatment in tracheotomized stroke patients: a randomized controlled trial. Intensive Care Med..

[59] Dziewas R, Stellato R, van der Tweel I (2018). Pharyngeal electrical stimulation for early decannulation in tracheotomised patients with neurogenic dysphagia after stroke (PHAST-TRAC): a prospective, single-blinded, randomised trial. Lancet Neurol..

